# Ca^2+^ monitoring in *Plasmodium falciparum* using the yellow cameleon-Nano biosensor

**DOI:** 10.1038/srep23454

**Published:** 2016-03-23

**Authors:** Kishor Pandey, Pedro E. Ferreira, Takeshi Ishikawa, Takeharu Nagai, Osamu Kaneko, Kazuhide Yahata

**Affiliations:** 1 Department of Protozoology, Institute of Tropical Medicine (NEKKEN), Nagasaki University, 1-12-4 Sakamoto, Nagasaki 852-8523, Japan; 2 Nepal Academy of Science and Technology (NAST), GPO Box: 3323, Khumaltar, Lalitpur, Nepal; 3 School of Biological Science, Nanyang Technological University, Singapore; 4 Life and Health Sciences Research Institute (ICVS), School of Health Sciences, University of Minho, Braga, Portugal; 5 Department of Molecular Microbiology and Immunology, Graduate School of Biomedical Sciences, Nagasaki University, 1-12-4 Sakamoto, Nagasaki 852-8523, Japan; 6 The Institute of Scientific and Industrial Research, Osaka University, 8-1 Mihogaoka, Ibaraki, Osaka 567-0047, Japan

## Abstract

Calcium (Ca^2+^)-mediated signaling is a conserved mechanism in eukaryotes, including the human malaria parasite, *Plasmodium falciparum*. Due to its small size (<10 μm) measurement of intracellular Ca^2+^ in *Plasmodium* is technically challenging, and thus Ca^2+^ regulation in this human pathogen is not well understood. Here we analyze Ca^2+^ homeostasis via a new approach using transgenic *P. falciparum* expressing the Ca^2+^ sensor yellow cameleon (YC)-Nano. We found that cytosolic Ca^2+^ concentration is maintained at low levels only during the intraerythrocytic trophozoite stage (30 nM), and is increased in the other blood stages (>300 nM). We determined that the mammalian SERCA inhibitor thapsigargin and antimalarial dihydroartemisinin did not perturb SERCA activity. The change of the cytosolic Ca^2+^ level in *P. falciparum* was additionally detectable by flow cytometry. Thus, we propose that the developed YC-Nano-based system is useful to study Ca^2+^ signaling in *P. falciparum* and is applicable for drug screening.

Malaria is caused by the intracellular protozoan parasite *Plasmodium*, and remains a major global public health problem^1^. The malaria parasite life cycle is complex and involves several cellular transformation events and multiplication stages, within both the vertebrate host and mosquito vector. The means by which the parasite recognizes and responds to its environment, and the cell signaling mechanisms which regulate progression through the life cycle, are not well understood. Because these signaling mechanisms and downstream pathways likely involve parasite-specific components, they are of interest for characterization in order to identify drug targets. The importance of new drug development is highlighted by the lack of an effective vaccine, and the observed development of parasite resistance against the present regimens of antimalarial drugs. Thus the development and deployment of antimalarial drugs remains an important strategy to target the liver, blood and transmission stages of the malaria parasite.

Malaria parasite infection begins with the inoculation of sporozoite stage parasites by the mosquito, followed by a period of parasite replication in the liver, and then release of merozoite stages into the blood to begin the intraerythrocytic cycle. It is the asexual repetition of red blood cell (RBC) invasion, development, multiplication via schizogony, and RBC rupture to release new merozoites which amplifies the blood stream parasite burden and provokes malarial symptoms and pathology. Some of the intra-erythrocytic parasites transform to non-replicative macro- and microgametocytes, which are the only stages transmissible to mosquitoes following the taking of a blood meal and which mediate sexual stage development in the mosquito midgut. The life cycle completes by the formation of sporozoites with midgut oocysts, and accumulation of these invasive sporozoites in the mosquito salivary gland. Herein we focus on the intraerythrocytic asexual cycle and gametocytes to characterize the storage and flow of calcium (Ca^2+^).

Calcium is a universal secondary messenger for intracellular signaling in eukaryotic cells and regulates a variety of cellular functions through fluctuation of cytosolic free Ca^2+^ 2. In malaria parasites Ca^2+^ has been implicated as a key second messenger and is maintained at a low level in the cytosol^3^. Ca^2+^ signaling has an essential role in *Plasmodium* cell differentiation, motility, egress from and invasion into the RBC in the blood stage parasites, as well as predicted roles in other lifecycle stages^4^. However, the mechanism of Ca^2+^ signaling in malaria parasite is not well understood^5^. Calcium signaling has been shown to trigger the activation of calcium-dependent protein kinases (CDPKs) and proteinases, which in turn stimulate parasite egress from the infected RBC (iRBC)^6^,^7^. During the cell invasion step, the secretion of adhesins from the apical microneme organelles is stimulated by Ca^2+^ through a phospholipase C (PLC) pathway in both *Plasmodium falciparum* and the distantly related apicomplexan parasite, *Toxoplasma gondii*^8^,^9^. Among several putative intracellular Ca^2+^ storage compartments in *P. falciparum*^10^, the endoplasmic reticulum (ER) is known to regulate cytosolic Ca^2+^ through the sarco/endoplasmic reticulum Ca^2+^-ATPase (*Pf*SERCA or *Pf*ATP6) pump^11^,^12^. Because SERCA has been studied as a target of therapeutic intervention in cancer^13^, and since *P. falciparum* genome contains only one SERCA gene^14^, targeting this essential pathway related to Ca^2+^ homeostasis in *P. falciparum* is an appealing approach to antimalarial drug development.

The cell biology of intracellular Ca^2+^ has been studied using synthetic chemical fluorescent Ca^2+^ indicators; however, these types of fluorochromes have drawbacks^15^. Specifically, because the fluorochromes occupy not only the cytosol, but also intracellular compartments such as ER, mitochondria and digestive food vacuole (DV), they are not suitable to evaluate Ca^2+^ concentration in a specific compartmentalized space in the eukaryotic cells including malaria parasites^16^. Malaria parasites possess numerous transporters which presumably localize on the membrane of such different intracellular compartments to actively transport chemical compounds across membranes^17^. For example, Fluo 4-AM is accumulated in the DV of *P. falciparum*, and the degree of accumulation appears to differ among parasite strains depending on the gene copy number encoding the transporter located on this membrane^18^. These fluorochromes exhibit a high dissociation constant value not only in mammalian cells but also in malaria parasites, and are therefore not suitable to evaluate the low static Ca^2+^ concentration of less than 100 nM which is proposed for *P. falciparum*^19^. Thus, the investigation of a cytosolic free Ca^2+^ concentration using chemical Ca^2+^ indicators must be approached with caution. To overcome these limitations, we consider here a recently designed genetically encoded Ca^2+^ indicator, yellow cameleon-Nano (YC-Nano) which is ultrasensitive and able to detect nanomolar changes of Ca^2+^ concentration in mammalian cell lines^20^. The YC-Nano Ca^2+^ biosensors were based on cyan fluorescent protein (CFP) fused with the Ca^2+^ binding protein, calmodulin (CaM), and yellow fluorescent protein (YFP) fused with M13 peptide. The CaM domain binds to M13 peptide in the presence of Ca^2+^, which in turn shortens the distance and thereby increases fluorescence resonance energy transfer (FRET) efficiency between the two fluorescent proteins. The advantage of the biosensor compared with fluorochromes is the nature of the output, which is the ratio of the emitted YFP signal to the emitted CFP signal. This ratio metric output reduces the artifacts introduced by unstable focusing due to parasite movement during live cell imaging process, which may occur with non-ratiometric indicators such as fluorochromes.

Here we report for *P. falciparum* the application of YC-Nano and the successful measurement of changes of cytosolic Ca^2+^. Live cell confocal microscopy revealed that cytosolic Ca^2+^ was maintained at a low concentration only at the trophozoite stage, and increased as intraerythrocytic development progressed. We show that the mammalian SERCA pump inhibitor thapsigargin (TG) and dihydroartemisinin (dART), a current first-line antimalarial, did not change the cytosolic Ca^2+^ concentration, indicating that these compounds do not inhibit *P. falciparum* SERCA pump activity. Docking analysis supported the insensitivity of the parasite to TG and dART. We also demonstrate detection of the FRET signal by a flow cytometry method, indicating that the transgenic reporter parasite is applicable for the high-throughput screening of compounds targeting *P. falciparum* Ca^2+^ homeostasis.

## Results

### Establishment and calibration of the Ca^2+^ biosensor YC-Nano in the malaria parasite *P. falciparum*

**Figure 1:**
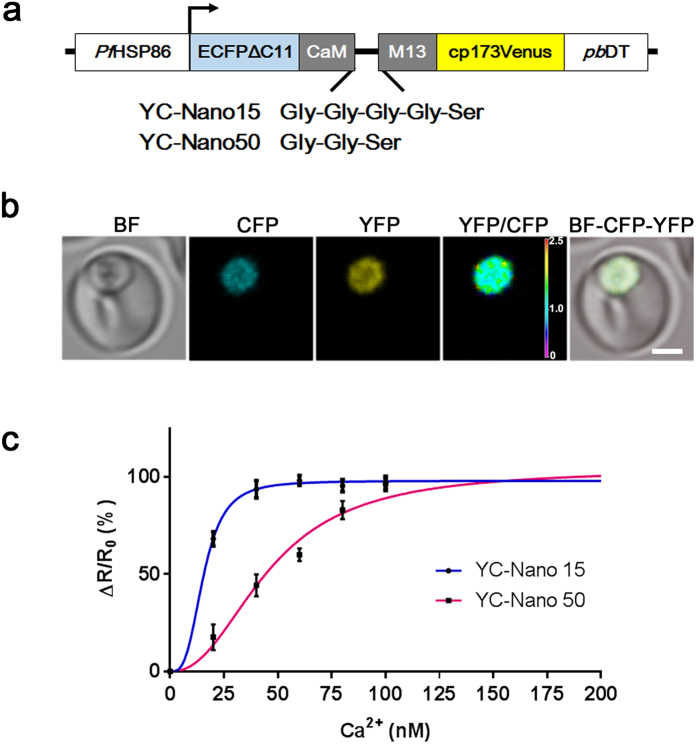
Design and properties of YC-Nano Ca^2+^ biosensors in *P. falciparum.* (**a**) Graphic representation of plasmid construction. Amino acid sequences of the linker between calmodulin (CaM) and myosin light chain (M13) peptide for YC-Nano15 and YC-Nano50 are shown. (**b**) Live cell images of the trophozoite stage parasite. Bright field (BF), CFP, YFP, and FRET (YFP/CFP) signals and merged image (BF-CFP-YFP). Purple to red color scale in the YFP/CFP panel represents low to high FRET efficiency (0 to 2.5). Scale bar, 2.5 μm. (**c**) The normalized fractional changes of the FRET signals (ΔR/R_0_) are plotted against the different Ca^2+^ concentration (0, 20, 40, 60, 80, and 100 nM). The curves represent the averaged data of ten parasites from 3 independent experiments.

To monitor the changes of cytosolic free Ca^2+^ in *P. falciparum*, we generated transgenic lines expressing fluorescent protein-based Ca^2+^ biosensors YC-Nano15 or YC-Nano50 driven by the *P. falciparum* heat shock protein 86 (*Pf*HSP86) constitutive promoter (Fig. 1a). The difference in the sensitivity to Ca^2+^ between these biosensors results from different lengths of the linker peptide between CaM domain and M13 peptide. The generated transgenic lines showed strong fluorescence signals in the parasite cytosol (Fig. 1b). In order to evaluate Ca^2+^ sensing capacity, we generated calibration curves using live trophozoite stages of these transgenic parasites. To exclude the indirect influence of Ca^2+^ in the iRBC cytosol and PV space, iRBC were treated with saponin to permeabilize the RBC and parasitophorous vacuole membrane (PVM) surrounding the parasite. The ratio of YFP and CFP was determined by confocal microscopy with Tyrode’s buffer containing different concentrations of Ca^2+^ (0–500 nM). The obtained calibration curves revealed a dissociation constant value of 15.5 nM and 45.8 nM for YC-Nano15 and YC-Nano50, respectively (Fig. 1c). These values are in agreement with the observed values by *in vitro* Ca^2+^ titration for those biosensors expressed in *Escherichia coli*; 15.8 nM and 52.5 nM, respectively^20^. Thus, our results indicate that malaria parasites efficiently express functional YC-Nano biosensors in the parasite cytosol. Because reports showed that the concentration of free Ca^2+^ in the parasite cytosol was roughly 40–100 nM by using synthetic chemical fluorescent Ca^2+^ indicator Fura 2^21^, consistent with our preliminary data using generated transgenic parasites, we selected YC-Nano50 for further experiments to monitor Ca^2+^ in *P. falciparum* cytosol, which has a more suitable dynamic range than YC-Nano15 for this purpose.

### Resting cytosolic Ca^2+^ concentration of intraerythrocytic *P. falciparum*

**Figure 2:**
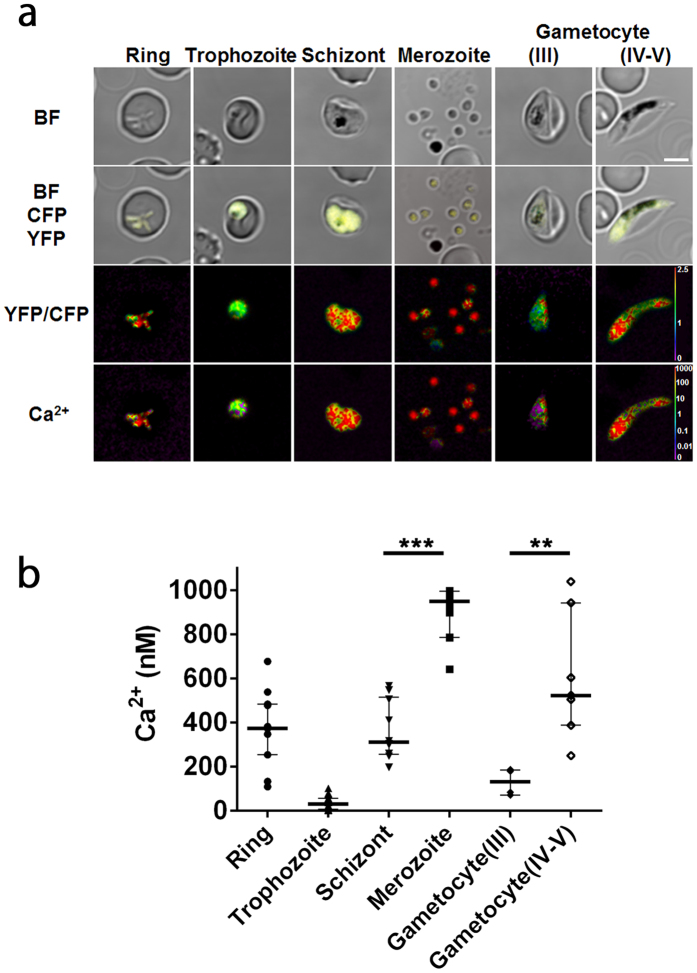
Cytosolic Ca^2+^ concentration in the different developmental stages of *P. falciparum.* (**a**) FRET signals from amoeboid ring (n = 11), trophozoite (n = 18), schizont (n = 10), merozoite (n = 10), and gametocyte (stage III (n = 6) and stage IV-V (n = 7)) stages of the parasite. Bright field (BF), merged image of BF, merged image (BF-CFP-YFP), FRET (YFP/CFP) signals, and calculated Ca^2+^ concentration with pseudo color are shown. Purple to red color scale in FRET (YFP/CFP) signals and calculated Ca^2+^ concentration represent low to high FRET efficiency (0 to 2.5) and 0 to 1000 nM Ca^2+^, respectively. Scale bar, 4 μm. (**b**) Calculated cytosolic Ca^2+^ concentrations of parasites with median and interquartile range are shown for each stage. **p = 0.0012, ***p < 0.0001 by Mann-Whitney *U*-test.

To describe the constitutive expression of the fluorescent proteins, we examined the YC-Nano50 fluorescence throughout the malaria parasite life cycle in RBC in addition to the trophozoite stage. We detected fluorescent signals from all blood stages (amoeboid ring, schizont and merozoite stages) and gametocytes, at higher FRET signals than in the trophozoite stage (Fig. 2a). To estimate the resting Ca^2+^ concentration of the parasite cytosol from FRET signals, we used the *in situ* Ca^2+^ calibration method with the Grynkiewicz equation^22^ (Supplementary Fig. S1). Addition of 10 mM Ca^2+^ containing the calcium ionophore A23187 to the Ca^2+^ free parasite culture increased YFP/CFP ratio from 1.36 (Ca^2+^ = 2.93 nM (median); minimum between 0–30 sec) to 2.66 (Ca^2+^ = 895.2 nM; maximum between 120–180 sec) in trophozoite stage parasites, confirming that YC-Nano50 has a large dynamic range in *P. falciparum.* The calculated median cytosolic Ca^2+^ concentration in the trophozoite stage was 30.0 (interquartile range: 5.6–55.0) nM. We found calculated Ca^2+^ concentrations in other stages of the parasite were much higher; 372.5 (253.0–483.0) nM at ring, 310.0 (256.2–514.9) nM at schizont, 949.6 (785.1–995.2) nM at merozoite, 131.3 (70.1–185.1) nM at gametocyte (stage III) and 521.8 (387.2–942.2) nM at gametocyte (stage IV–V) stages (Fig. 2b).

### *P. falciparum* cytosolic Ca^2+^ level is not modulated by thapsigargin, a mammalian SERCA inhibitor

**Figure 3:**
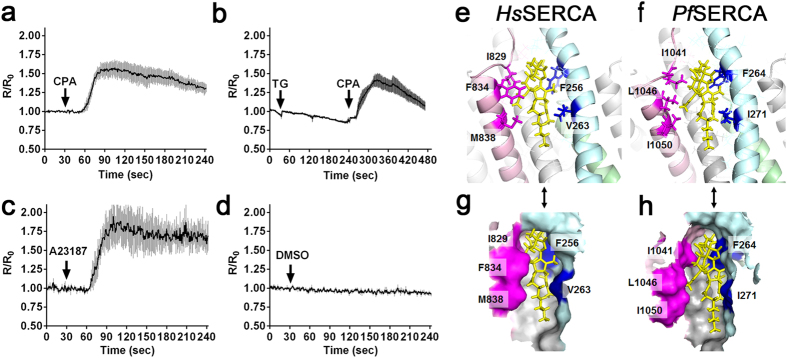
Effect of cyclopiazonic acid (CPA) and thapsigargin (TG) on cytosolic Ca^2+^ levels in *P. falciparum* and their docking models. (**a**) Time course of the cytosolic Ca^2+^ level with the addition of 15 μM CPA at 30 sec (arrow). The traces were generated from the mean and standard error of the mean. (n = 4). (**b**) Time course of the cytosolic Ca^2+^ level with the addition of 7.6 μM TG at 30 sec (arrow) and 15 μM CPA at 240 sec (arrow, n = 5) (**c**) Time course of the cytosolic Ca^2+^ level with the addition of 10 μM A23187 at 30 sec (arrow, n = 3) (**d**) Time courses of the cytosolic Ca^2+^ level with the addition of DMSO as a solvent control at 30 sec (arrow, n = 3). The cytosolic Ca^2+^ level is represented by R/R_0_ value, where R is the YFP/CFP ratio and R_0_ is the mean YFP/CFP ratio before addition of the drug as baseline (time between 0–30 s). Model structures of TG (yellow) with human SERCA (*Hs*SERCA) (**e**) or *P. falciparum* SERCA (*Pf*SERCA) (**f**) Illustrate a structural difference in the TG-binding pocket. Colored regions of each SERCA are located within 4.0 Å distance from TG and were used for binding energy calculations. (**g**,**h**) Schematics represent surface structure from ribbon diagrams corresponding to (**e**,**f**), respectively.

The endoplasmic reticulum is an important Ca^2+^ storage compartment to maintain and regulate the cytosolic Ca^2+^ concentration in eukaryotic cells, and uptake of Ca^2+^ from cytosol to ER is regulated by SERCA. In *Plasmodium* conflicting reports describe the responses of malaria parasites against SERCA inhibitors, specifically thapsigargin (TG)^21^,^23^. We therefore revisited the effect of TG for parasite cytosolic Ca^2+^ homeostasis, and found that 15 μM CPA, a SERCA specific inhibitor consistently reported to inhibit *P. falciparum* SERCA (*Pf*SERCA)^24^, increased the cytosolic Ca^2+^ (Fig. 3a); whereas 7.6 μM TG, a concentration reported to inhibit *Pf*SERCA pump activity^23^, did not change the cytosolic Ca^2+^ concentration (Fig. 3b). The effect of TG on the cytosolic Ca^2+^ level was not observed even when 76 μM TG was applied (Supplementary Fig. 2). The positive control calcium ionophore A23187 increased the cytosolic Ca^2+^, and a solvent control DMSO showed no effect (Fig. 3c,d).

Because the parasite is surrounded by Ca^2+^ rich environments in the human body and in the culture - for example, 45–86 nM in the RBC cytosol, ~40 μM in the PV space, and ~1 mM in the human plasma^25^,^26^ - we further evaluated the effect of CPA and TG in Ca^2+^-free medium after selective membrane permeabilization. Firstly, iRBCs were treated with streptolysin O (SLO) to selectively permeabilize the RBC membrane, but not the PVM and parasite plasma membrane (PPM). When TG was added to SLO-treated iRBC, no effect was observed, but subsequent addition of CPA increased the cytosolic Ca^2+^ (Supplementary Fig. S3a), consistent with the previous result, thus indicating that the observed effect of CPA and TG was not due to Ca^2+^ in the RBC cytosol or medium. Next, iRBCs were treated with saponin to permeabilize the RBC membrane and PVM, but not the PPM. Again CPA increased cytosolic Ca^2+^, but TG did not (Supplementary Fig. S3b), indicating the effect of CPA and TG was not due to Ca^2+^ in the PV space. These results confirmed that the parasite cytosolic Ca^2+^ concentration changed in response to CPA in the presence or absence of RBC membrane and parasitophorous vacuole membrane, indicating that CPA targeted intracellular Ca^2+^ storage.

To gain insights into the difference between human SERCA and *Pf*SERCA in the response against TG, we constructed a model structure of human SERCA and *Pf*SERCA based on the co-crystal structure of rabbit SERCA with TG using homology modeling. Docking simulation with 200 individual genetic algorithm from homology modeling resulted in an estimated binding free energy and inhibitory constant (*K*_*I*_) for TG and *Pf*SERCA of −9.58 kcal/mol and 95.62 nM, respectively; whereas those for TG and human SERCA were −10.82 kcal/mol and 11.77 nM, respectively. A more negative free energy value indicates a stronger molecular interaction, and thus the results suggest a 8.1-fold weaker interaction between *Pf*SERCA and TG than that between human SERCA and TG. Next, to optimize the interaction between protein and TG in detail, we performed energy minimizations from homology modeling, then we made an energy minimized structure of complex of human SERCA and *Pf*SERCA with TG (Fig. 3e–h). Interaction energy was calculated as the van der Waals force between TG and the 34 amino acids located within 4.0 Å distance from TG (Supplementary Figs S4 and S5). Five amino acid residues (F_256_, V_263_, I_829_, F_824_, and M_838_ for human SERCA versus F_264_, I_271_, I_1041_, L_1046_ and I_1050_ for *Pf*SERCA) at the same position in the SERCA structure indicated a difference of TG binding pocket and binding energy. For example, L_1046_ of *Pf*SERCA occupied the location where F_834_ of human SERCA exists, which results in more open space in the TG-binding pocket of *Pf*SERCA. The total value between *Pf*SERCA and TG was −99.0, a value higher than that between human SERCA and TG (−100.8). Because a more negative value of the estimated van der Waals force indicates more stability, these results also suggest that the interaction between *Pf*SERCA and TG is less stable than that between human SERCA and TG. These homology modeling and binding energy calculations between *Pf*SERCA and TG support the hypothesis of *P. falciparum* insensitivity against TG.

### Dihydroartemisinin does not alter the cytosolic Ca^2+^ homeostasis of *P. falciparum*

**Figure 4:**
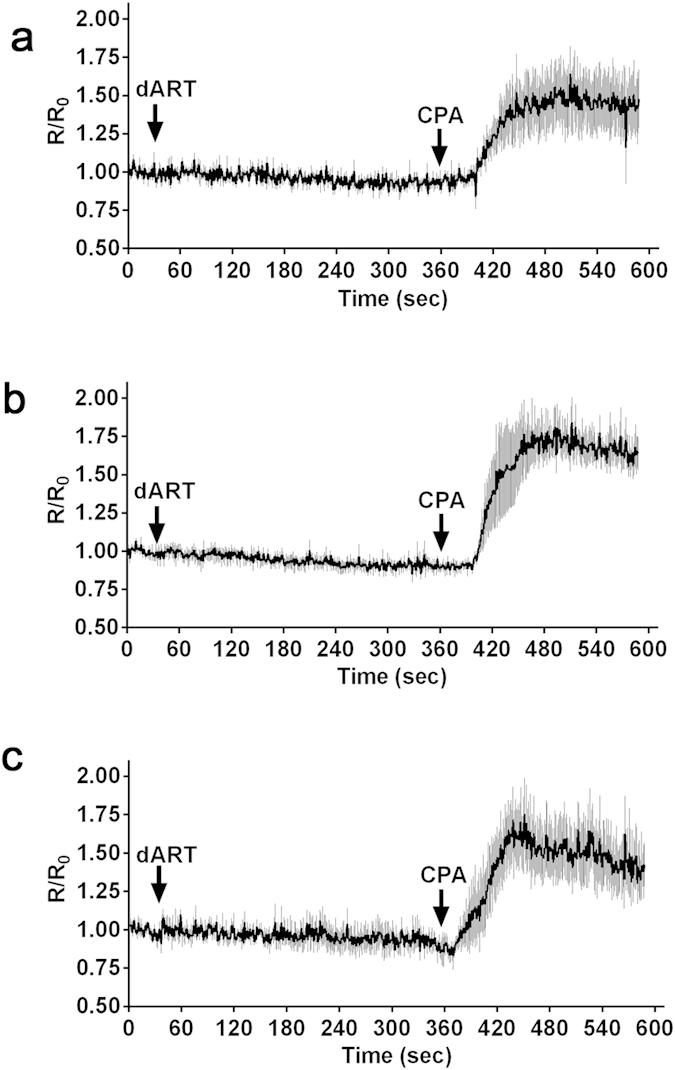
Effect of different concentrations of dihydroartemisinin (dART) on the cytosolic Ca^2+^ level in *P. falciparum.* Time course of the cytosolic Ca^2+^ level with the addition of 1 (**a**), 10 (**b**), and 100 μM (**c**) dART at 30 sec (arrow) and 15 μM cyclopiazonic acid (CPA) at 360 sec (arrow). The trace was generated from the mean and standard error of the mean of at least 3 independent experiments.

Artemisinin (ART) derivatives are currently the most commonly used anti-malarial drugs, due to their low cost and because *P. falciparum* has not developed resistance against these drugs outside of Southeast Asia^1^. In spite of their importance for treatment of malaria, the mechanism of action of the active metabolite of the ART derivatives, dihydroartemisinin (dART), in the malaria parasite is still not clearly understood^27^. Two mechanisms of action have been proposed, the first that dART targets parasite hemoglobin metabolism^28^ and the second that dART targets SERCA^29^. Because both dART and TG are sesquiterpene lactones and might act towards SERCA in a similar manner, we evaluated if dART could disturb cytosolic Ca^2+^ homeostasis as implicated from the latter hypothesis. We exposed parasites to different concentration of dART (1, 10, and 100 μM) and found that dART had no effect on the parasite cytosolic Ca^2+^ concentration even at 100 μM (Fig. 4a–c). Sequential exposure of the dART-treated parasites to CPA increased cytosolic Ca^2+^, thus validating that the parasites were responsive to inhibitors. The IC_50_ of dART used in the above experiment against *P. falciparum* was 1.5 nM for 24 hours^30^, confirming the pharmacological activity of dART. These results suggest that dART does not target *Pf*SERCA.

Docking simulation of dART with *Pf*SERCA or human SERCA were performed with 200 individual genetic algorithm from homology modeling resulted that the estimated free energy and *K*_*I*_ for *Pf*SERCA and dART were −6.96 kcal/mol and 7.85 μM, respectively. For human SERCA and dART the values for estimated free energy and *K*_*I*_ were −7.47 kcal/mol and 3.37 μM, respectively; suggesting, firstly, a 2.3-fold weaker interaction between *Pf*SERCA and dART than that between human SERCA and dART; and, secondly, a much weaker interaction of dART with both human and *Pf*SERCA than the case for TG. These modeling calculations support the observed inactivity of dART against cytosolic Ca^2+^ homeostasis in *P. falciparum*.

### Ca^2+^ is stored in compartments other than the ER at the trophozoite stage of *P. falciparum*

**Figure 5:**
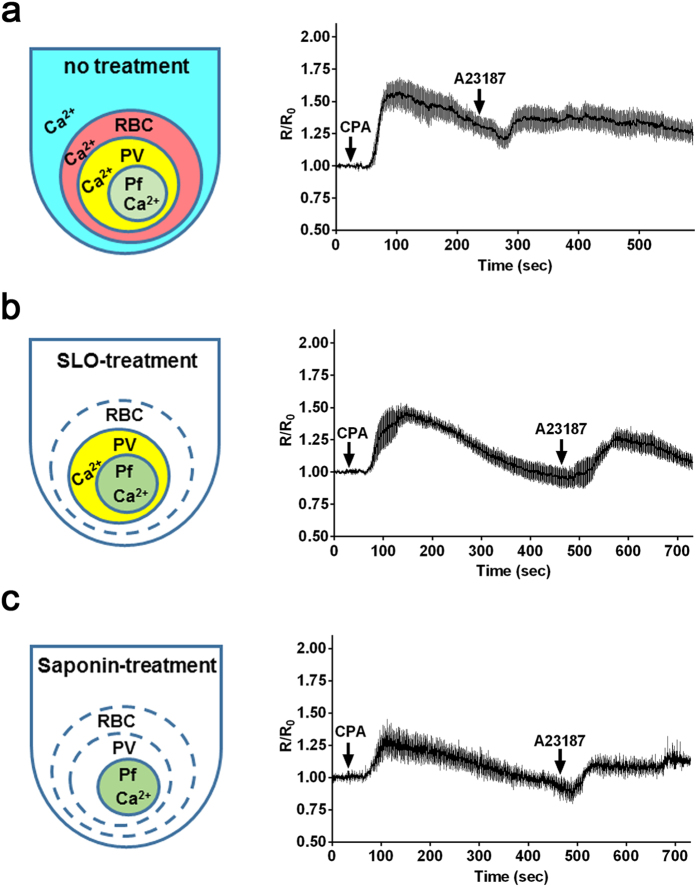
Effect of the membrane permeabilization of the parasite-infected red blood cells on the cytosolic Ca^2+^ level in *P. falciparum.* Schematic indicates the status of the existence of Ca^2+^ in the different compartments in the parasite-infected red blood cell (iRBC) without treatment (**a**), treated with streptolysin O (SLO) (**b**), or treated with saponin (**c**). Time courses of the cytosolic Ca^2+^ level with the addition of 15 μM cyclopiazonic acid (CPA) and 10 μM A23187 to non-treated iRBCs in Ca^2+^-containing RPMI medium (**a**), with the addition of 3 μM CPA and 2 μM A23187 to SLO-treated iRBCs in Ca^2+^ free medium (**b**), or with the addition of 3 μM CPA and 2 μM A23187 to saponin-treated iRBCs in Ca^2+^ free medium (**c**). A lower concentration of CPA and A23187 was used to avoid the damage to the SLO-treated and saponin-treated iRBCs. The traces are generated from the mean and standard error of mean of 4 independent experiments.

In the present study we detected an increase of cytosolic Ca^2+^ level in the Ca^2+^-containing medium with the calcium ionophore A23187, but not CPA, even after SERCA was inhibited with CPA as a SERCA specific inhibitor (Fig. 5a, Supplementary Fig. S6a), thereby indicating that the second peak of Ca^2+^ level was not derived from the ER. In contrast, addition of CPA to the A23187-pretreated iRBCs in the Ca^2+^-containing medium did not increase the cytosolic Ca^2+^ (Supplementary Fig. S6b). Although the ER is the main site of intracellular Ca^2+^ storage, other intracellular compartments are known to contribute to this process in the phylum Apicomplexa^4^, and therefore these results suggest that Ca^2+^ flows into the parasite cytosol either from other Ca^2+^-storing compartments or from outside of the parasite. To investigate if we could detect the existence of non-ER Ca^2+^ storage sites using the transgenic reporter parasites, we excluded the Ca^2+^ source in the iRBC cytosol and medium. iRBC were selectively permeabilized with SLO or saponin and the effect on the cytosolic Ca^2+^ level was evaluated by sequential addition of CPA and A23187 in the Ca^2+^ free medium. When A23187 was added to SLO-treated and CPA-pretreated iRBC, cytosolic Ca^2+^ was increased (Fig. 5b), suggesting that second elevation of Ca^2+^ level was not due to the influx from medium or RBC cytosol. Next, to exclude the Ca^2+^ source in the PV space, cytosolic Ca^2+^ level was evaluated using saponin-treated iRBC in the Ca^2+^ free medium. Addition of A23187 still increased the cytosolic Ca^2+^ level in the CPA-pretreated parasites (Fig. 5c), indicating that the second elevation of Ca^2+^ was not due to influx from the PV. Furthermore, we found that the second peak of cytosolic Ca^2+^ in the parasites treated with saponin was significantly lower than the peak with SLO (13% reduction; n = 4; p < 0.0001, Mann–Whitney *U*-test), suggesting that this reduction reflect Ca^2+^ influx from PV into parasite cytosol in SLO-treated iRBC. These results indicate that our system is able to detect the existence of parasite Ca^2+^ in the PV space and intracellular Ca^2+^ storage compartments in addition to the ER.

### Flow cytometry-based system is applicable for drug screens targeting *P. falciparum* Ca^2+^ homeostasis

**Figure 6:**
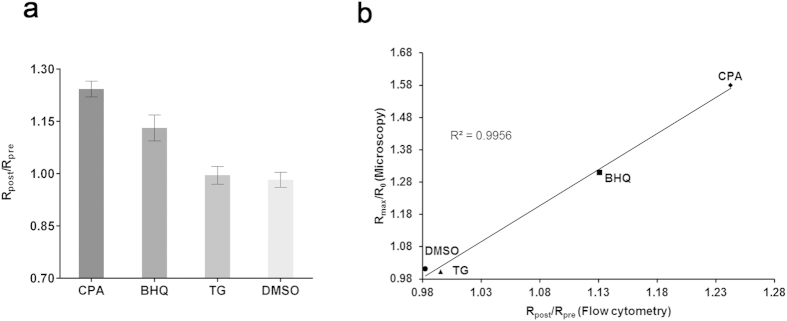
Detection of the cytosolic Ca^2+^ change in *P. falciparum* by flow cytometry. (**a**) FRET signals are represented by R_post_/R_pre_ where R_pre_ is the YFP/CFP ratio before addition of inhibitors and R_post_ is the YFP/CFP ratio after addition of inhibitors. The mean and standard error of the mean of R_post_/R_pre_ were obtained with 15 μM cyclopiazonic acid (CPA), 2 μM 2,5-Di-t-butyl-1,4-butylhydroquinone (BHQ), 7.6 μM thapsigargin (TG) and DMSO from 3 independent experiments. (**b**) Correlation of FRET signal values obtained by confocal microscopy and the values by flow cytometry. Maximum of FRET signal changes (R_max_/R_0)_ from microscopic analysis and the FRET signal changes (R_post_/R_pre_) by flow cytometry are plotted. Linear regression line and the coefficient of determination (R^2^ = 0.9956) are shown.

In order to develop a high-throughput method to screen panels of compounds, we examined if flow cytometry could be used to detect FRET signals in the YC-Nano50-expressing *P. falciparum* (Fig. 6a). In this assay we included another SERCA inhibitor, 2,5-di-*tert*-butylhydroquinone (BHQ), in addition to CPA and TG, to compare the FRET signals obtained by confocal microscopy (Fig. 3a,b and Supplementary Fig. 6c) and those by flow cytometry. BHQ is known for its structural simplicity and low cost in comparison to other SERCA inhibitors^31^. The R_post_/R_pre_ values by flow cytometry were 1.24 ± 0.02 (mean ± standard error of the mean (s.e.m.)), 1.13 ± 0.03, 0.99 ± 0.02, and 0.98 ± 0.02, for CPA, BHQ, TG, and DMSO, respectively (Fig. 6a); indicating that CPA and BHQ, but not TG, affect the cytosolic Ca^2+^ homeostasis. In the above experiments the IC_50_ values against *P. falciparum* for CPA, BHQ, and TG were 1.1 ± 0.06 (mean ± s.e.m.), 0.26 ± 0.01, and 32.8 ± 4.8 μM, respectively; thus validating the pharmacological activity of all compounds (Supplementary Table). Clear correlation (R^2^ = 0.9956) existed between FRET signals obtained from confocal microscopy and flow cytometry (Fig. 6b).

## Discussion

In this study we generated transgenic *P. falciparum* lines which stably express genetically encoded YC-Nano Ca^2+^ biosensors in the cytosol, and a robust system to monitor Ca^2+^ concentrations under physiological conditions. This technology enabled us to evaluate the cytosolic Ca^2+^ concentration of the parasite cytosol at different developmental stages, and to monitor the change in cytosolic Ca^2+^ levels caused by a panel of compounds proposed to act against the ER-residing Ca^2+^-ATPase, SERCA. As an initial attempt, to avoid cell damage and obtain reproducible FRET signals without photobleaching, we used a 1% (<3 μW) power of 457 nm laser beams for excitation. Introduction of the Perfect Focus System and galvano scanner enabled stable capture images every 1 second at a 512 × 512 pixel resolution, which is critical to monitor changes in organisms of sizes less than 10 μm diameter, such as the malaria parasite. With these optimizations the FRET signals from this organism became stable for 10 minutes or more.

**Figure 7:**
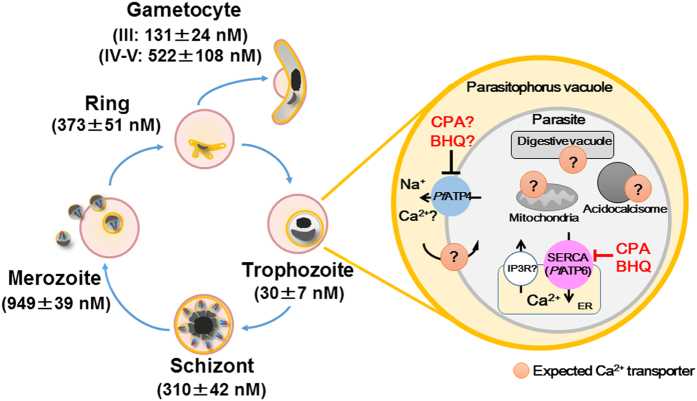
Schematic illustration of the cytosolic Ca^2+^ homeostasis in *P. falciparum*. Blood stage parasites observed in *in vitro* cultures and their estimated cytosolic Ca^2+^ concentrations are indicated. In the trophozoite stage parasite, *P. falciparum* SERCA (*Pf*ATP6) is inhibited by cyclopiazonic acid (CPA) and 2,5-Di-t-butyl-1,4-butylhydroquinone (BHQ), but not by thapsigargin (TG) and dihydroartemisinin (dART). CPA and BHQ may also affect *Pf*ATP4, a Na^+^-ATPase located on the parasite plasma membrane (PPM), and inhibit Ca^2+^ influx from parasite cytosol to parasitophorous vacuole. Expected non-ER Ca^2+^-containing compartment(s) with potential integral membrane Ca^2+^ transporters are indicated. Inositol trisphosphate receptor (IP_3_R) on the ER membrane has been proposed, but the encoding gene has not been identified^4^.

To our knowledge this is the first report to estimate cytosolic Ca^2+^ concentrations throughout the blood stages of the malaria parasite. The cytosolic Ca^2+^ concentration is high for all stages (values for amoeboid ring, schizont, merozoite, gametocyte stage III and gametocyte stage IV-V are 373, 310, 949, 131 and 522 nM, respectively), with the exception of the trophozoite (30 nM) (Fig. 7). The trophozoite is metabolically the most active stage, which may favor a lower cytosolic Ca^2+^ in order to respond to subtle changes in Ca^2+^ concentrations. The trophozoite stage parasite is able to quickly recover the cytosolic Ca^2+^ level after the artificial increase of the Ca^2+^ level with SERCA inhibitors (Fig. 5), indicating the existence of the SERCA-independent mechanisms to maintain the cytosolic Ca^2+^ level less than 100 nM. *Pf*ATP4 (PF3D7_1211900), a non-SERCA-type Ca^2+^-transporting P-ATPase which is located on the parasite plasma membrane, may participate to this process^32^. Because the cytosolic Ca^2+^ level significantly increased in the schizont stage, both mechanisms appear to be less active in this mature parasite form. The estimated cytosolic Ca^2+^ concentration was highest at the merozoite stage, significantly higher than the schizont stage (n = 10; p < 0.0001, Mann-Whitney *U*-test). Ca^2+^ signaling is known to be involved in the egress of the merozoites from the RBC, as well as the invasion into new RBC by triggering the secretion of microorganelles such as exonemes and micronemes in the merozoite stage parasite^7^,^9^. Thus we consider that the observed highest cytosolic Ca^2+^ level at released merozoites indicates that the Ca^2+^ secretion signals have been initiated. Glushakova *et al.* reported that cytosolic Ca^2+^ level increased at the schizont stage, reaching to 1–10 μM range just prior egress using a chemical indicator Fura Red, which is consistent to our estimated cytosolic Ca^2+^ concentration of 949 nM at the merozoite stage^33^. *P. falciparum* calcium-dependent protein kinase 5 (*Pf*CDPK5) and *Pf*CDPK1 are plant-like protein kinase family members and have been proposed to act during these steps^34^. Although it is not clear if these *Pf*CDPKs are activated at Ca^2+^ concentrations as high as the 310 nM estimated at the schizont stage, one report proposed that the *K*_*d*_ value for Ca^2+^ of a purified beetroot CDPK is 770 nM^35^. In this regard, Carey *et al.* successfully observed Ca^2+^ oscillation during *P. berghei* sporozoite movement using another calcium biosensor, TN-XXL, which has a *K*_*d*_ of 830 nM^36^. The higher Ca^2+^ level in the released merozoites was noted by Biagini *et al.* using Fluo 4-AM, but the concentration was not determined due to the limited resolution of the system^37^. After completing RBC invasion, the parasite appears to gradually establish mechanisms to regulate cytosolic Ca^2+^ level less than 100 nM during ring stage development. Because CDPK1 is also expressed in both male and female gametocytes^38^, high Ca^2+^ concentration estimated at the gametocyte stage is consistent with possible CDPK1 activity in the high Ca^2+^ level environment.

To validate the robustness of our established system to monitor the Ca^2+^ concentration in *P. falciparum*, we conducted four experiments to evaluate: 1) the effect of TG on the *P. falciparum* cytosolic Ca^2+^ homeostasis, 2) the effect of dART on the *P. falciparum* cytosolic Ca^2+^ homeostasis, 3) the feasibility to detect Ca^2+^ storage(s) beside ER, and 4) the feasibility to establish a high-throughput method to detect the change of the Ca^2+^ level by flow-cytometry. Uptake of Ca^2+^ from cytosol to ER is largely regulated by SERCA; however, there are conflicting observations regarding malaria parasite responses against the SERCA inhibitor TG. One report concluded that *Pf*SERCA was TG-insensitive^21^, but another reported TG-sensitivity^23^. This controversy may be in part due to the employed method to monitor the cytosolic Ca^2+^ with synthetic chemical indicators Fura 2-AM or Fluo 3-AM. Our analysis using a parasite line expressing a biosensor revealed that the Ca^2+^ concentration at the trophozoite stage was 30 nM, which was much lower than the dissociation constant of Fura 2- or Fluo 3-based indicators (*K*_*d*_ = 140 and 325 nM, respectively). Using the YC-Nano50 biosensor with a *K*_*d*_ value of 45.8 nM, which is superior than the chemical indicators to evaluate Ca^2+^ concentration at the trophozoite stage of *P. falciparum*, we were able to clarify that TG had no effect on the Ca^2+^ homeostasis of *P. falciparum*. SERCA of apicomplexan parasites, including *Plasmodium*, is evolutionally more closely related to one of the two types of plant SERCA than to mammalian SERCA^39^. TG is a plant-derived compound and the plant Ca^2+^-ATPases have developed insensitivity to TG^40^, which is in agreement to our observation that *Pf*SERCA is TG-insensitive. Docking models of TG with *Pf*SERCA and mammalian SERCA also suggest a clear difference in the shape of the TG binding pocket between the two SERCA structures. Based on these differences in the sensitivity against TG and the structure of the TG binding pocket, TG may serve as a seed compound for a structure-based drug design to develop selective anti-malarial compounds.

Because both TG and ART are composed of sesquiterpene lactone, and since TG is a highly selective inhibitor for mammalian SERCA^41^, it was therefore reasoned that both TG and ART would behave in a similar manner towards SERCA. Consistent to this expectation, studies analyzed Ca^2+^-ATPase activity using *Pf*SERCA expressed on *Xenopus* oocyte membrane proposed that ART had effect on *Pf*SERCA^42^,^43^. However, other experiments did not support that *Pf*SERCA was a target of ART^44^,^45^. In this study, we clearly showed that dART had no effect on Ca^2+^ concentration in the parasite cytosol. Docking models of dART and both SERCA showed that the affinity of dART to both SERCA is at the micromolar level, suggesting that dART may not be effective against both SERCA. Together, our data indicate that dART plays at most a minor role to modulate *P. falciparum* Ca^2+^ homeostasis.

The ER is the most important organelle storing Ca^2+^ in the malaria parasite^12^,^21^; but other compartments, such as DV^37^, mitochondrion^46^, acidocalcisome^10^, and PV space^47^ have also been proposed to act as Ca^2+^ storage sites (Fig. 7). In this study we indicate the existence of Ca^2+^ in the *P. falciparum* PV space by comparing SLO-treated iRBC and saponin-treated iRBC. In *P. falciparum*, two Ca^2+^ ATPases, *Pf*SERCA (*Pf*ATP6) and *Pf*ATP4, have been annotated among the 13 P-type ATPases. *Pf*ATP4 is localized on the PPM and is considered to transport not only Na^+^ but also Ca^2+^ 32,48. This ATPase is potentially responsible for the difference in the observed higher level of the cytosolic Ca^2+^ increase after calcium ionophore stimulation in SLO-treated iRBC than that in saponin-treated iRBC. The DV was reported to contain only moderate amounts of Ca^2+^ and no dynamic changes of the Ca^2+^ concentration were observed in DV following induced cytosolic Ca^2+^ bursts^37^. Although there are some reports of the mitochondria and acidocalcisome as Ca^2+^ storages, active participation of these compartments to maintain cytosolic Ca^2+^ homeostasis of malaria parasites is still unclear^49^.

In conclusion, we generated a transgenic *P. falciparum* expressing YC-Nano50 biosensor and showed that this parasite is a suitable and powerful tool in which to study Ca^2+^ homeostasis in the trophozoite stage of *P. falciparum.* We determined, for the first time, that the resting Ca^2+^ concentrations at schizont, merozoite, ring, and late gametocyte stages are higher than 300 nM. We also showed that TG and dART did not affect the cytosolic Ca^2+^ level of trophozoite stage of this parasite. FRET signals are detectable by flow cytometry and correlate with a microscope-based assay, indicating that the developed flow cytometry-based system is applicable for drug screens targeting mechanisms which maintain *P. falciparum* Ca^2+^ homeostasis.

## Methods

### Chemicals

Thapsigargin (TG), cyclopiazonic acid (CPA), calcium ionophore A23187, 2,5-Di-t-butyl-1,4-butylhydroquinone (BHQ), and dimethyl sulfoxide (DMSO) were purchased from Sigma Aldrich Chemical Co (St. Louis, USA). Dihydroartemisinin (dART) was purchased from Tokyo chemical industry Co (Tokyo, Japan). Stock solutions of all drugs were dissolved in DMSO.

### Generation of expression plasmids for Ca^2+^ biosensor

Plasmids for *P. falciparum* transfection were constructed based on the Invitrogen Multisite Gateway^®^ system (Invitrogen, Carlsbad, CA). DNA fragments encoding YC-Nano15 and -50 were amplified from corresponding plasmid templates by PCR amplification and recombined with pDONR™P2R-P3 to generate pENT23-YC-Nano15 and -50, respectively. Expression vectors, pLN-YC-Nano15 and 50 (Fig. 1a), were generated by LR reaction from pENT23 plasmids described above, pENT41 plasmid containing *P. falciparum* HSP86 promoter region, pENT12-linker, and pLN-DEST-R43(II) containing a blasticidin-s deaminase (BSD) selectable marker^50^. Nucleotide sequence data reported are available in the DDBJ Sequenced Read Archive under the accession numbers LC028929 and LC075581. All experiments conducted in this study were approved by the committee for recombinant DNA experiment, Nagasaki University. The methods were carried out in accordance with the approved guidelines.

### Parasite lines, culture, and transfection

The *P. falciparum* Dd2 parasite line was originally obtained from National Institute of Health, USA. The parasites were maintained with O^+^ human RBC at 2% hematocrit in fibrinogen-free human plasma-containing complete RPMI medium and transfection was performed as described^51^. At days 4–5 post transfection, drug selection with 2.5 μg/mL BSD (InvivoGen, San Diego, CA) was started and culture was maintained until drug-resistant parasites appeared. The usage of human RBC and plasma was approved by the ethical committee, Institute of Tropical Medicine, Nagasaki University.

### Cytosolic Ca^2+^ measurements

YC-Nano-expressing *P. falciparum* parasites (3–6% parasitemia) were used for live cell imaging experiments. Ca^2+^ measurements were performed using trophozoite parasites which were obtained by 5% sorbitol synchronization before 18–24 hr experimentation^48^. On the day of imaging, parasite cultures were collected and washed twice with 1 ml of 37 °C warmed plasma-free incomplete RPMI medium (ICM). Then 1 ml of 0.25% hematocrit parasite infected-RBC (iRBC) was plated on the glass bottom 35-mm cellview^TM^ TC treated hydrophilic coated dish (Grenier bio-one, Germany). After keeping the iRBC in the dish for 30 min, ICM were replaced with phenol red- and plasma-free RPMI medium containing 0.5% AlbuMAX^®^ I. Time-lapse imaging was performed at 37 °C using an A1R confocal microscope system configured with an inverted microscope (Ti-E; Nikon, Japan) with 60× or 100× oil objective lens (PlanApo, NA 1.4, Nikon). The inverted microscope configuration acts as a stable system with the Perfect Focus System (PFS, Nikon). The water chamber stage and the objective lens were kept at 37 °C with a temperature controller (Tokai-Hit, Japan). The fluorescence resonance energy transfer (FRET) image analysis between cyan fluorescent protein (CFP) and yellow fluorescent protein (YFP) was performed by confocal microscopy. YC-Nano was excited at 457 nm for both CFP and YFP, and emissions were detected for CFP (482/35 nm) and YFP (525/50 nm). Time-lapse images were captured every 1 sec at a 512 × 512 pixel resolution by confocal microscopy. For the first 30 sec, time-lapse images were taken without chemical compounds. Chemical compounds (TG, CPA, dART, A23187, BHQ, and DMSO control) were added directly to the edge of the chamber containing transgenic parasites. The parasite cytosolic region was used for the analysis as a region of interest (ROI) and background fluorescence was subtracted. The imaging analysis was carried out using NIS-Element Advanced Research imaging software (Nikon). The R/R_0_ value was calculated for each parasite, where R is the YFP/CFP ratio and R_0_ is the mean YFP/CFP ratio before adding the drug as baseline (time between 0–30 s).

### Permeabilization of parasite-iRBC with streptolysin O or saponin

To selectively permeabilize the RBC membrane only, iRBC were treated with 20 U/ml streptolysin O (SLO; Sigma Aldrich Chemical Co, St. Louis, MO) in PBS for 6 min at room temperature, washed three times with ICM, and kept in ICM^52^. To permeabilize RBC and PVM, iRBCs were treated with 0.01% saponin (Wako Pure Chemical Industries, Ltd, Japan) in PBS for 10 min at room temperature, washed three times with PBS, and kept in ICM^53^. SLO- or saponin-treated iRBCs were transferred to hydrophilic-coated dish and kept for 30 min to let the iRBCs adhere to the glass dish bottom. The iRBCs were washed three times for 10 min each with Ca^2+^ free Tyrode’s buffer (140 mM NaCl, 10 mM glucose, 10 mM HEPES, 4 mM KCl, 1 mM MgCl_2_, pH 7.4) to remove Ca^2+^ from the extracellular medium. Finally, Ca^2+^ free Tyrode’s buffer was used for time lapse imaging.

### YC-Nano calibration curve for *P. falciparum*

To generate the calibration curve, iRBCs were permeabilized with saponin and prepared for analysis as described above. Tyrode’s buffer containing different concentration of Ca^2+^ (0, 10, 20, 40, 60, 80, 100, and 500 nM) were prepared with calcium chloride. Parasites were re-suspended in the different concentrations of Ca^2+^-containing buffer, kept for 10 min, and observed under the confocal microscope. Images were obtained from 10 independent parasites for each Ca^2+^ concentration and the fractional change of the YFP to CFP ratio (ΔR/R_0_ where ΔR = R − R_0_) was calculated. The ΔR/R_0_ values were normalized by dividing by the ΔR/R_0_ value with 500 nM Ca^2+^ buffer and plotted using GraphPad Prism6 software (GraphPad Software, Inc., La Jolla, CA).

### Calculation of the resting cytosolic Ca^2+^ concentration

To estimate the resting cytosolic Ca^2+^ concentration in the different stages of *P. falciparum*, we employed an *in situ* Ca^2+^ calibration method with time course change of Ca^2+^ concentration in the individual intact iRBC, which is more precise than single images of the parasite^54^. The area of the parasite desired for quantification of the fluorescence signal, and the control area were selected as ROIs. YFP/CFP was determined in a given time course. The YFP/CFP value was converted to calcium concentration value using the following equation^22^. Concentration of free ionized Ca^2+^ 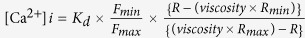 where *K*_*d*_ is a dissociation constant (45.8 nM for YC-Nano50 from Fig. 1), the viscosity is 1 in Ca^2+^ free Tyrode’s buffer or plasma-free RPMI medium containing 0.5% AlbuMAX^®^ I. R_min_ and R_max_ are the minimum or maximum YFP/CFP values obtained from trophozoite stage parasites with 1 mM EGTA and 10 μM A23187 (Ca^2+^-free condition) or with 10 mM CaCl_2_ and 10 μM A23187 (Saturated Ca^2+^ condition), respectively. F_min_ and F_max_ are the fluorescence intensities at 458 nm excitation at the moment of R_min_ or R_max_. Obtained R_min_, R_max_, F_min_, and F_max_ values were 1.36, 1233, 2.66, and 503, respectively. The different stages of *P. falciparum* were assayed in plasma-free RPMI medium containing 0.5% AlbuMAX^®^ I. The acquisition of FRET images and the calculation of the resting cytosolic Ca^2+^ concentration were performed using NIS-Element Advanced Research imaging software (Nikon) with the above formula. For example, Ca^2+^ concentration of one schizont was calculated as 345.6 nM using above equation from YFP/CFP value of 2.34. Obtained Ca^2+^ concentration using Grynkiewicz equation with above condition was limited to 1000 nM to avoid unacceptably large fluctuations by NIS-Element Advanced Research imaging software.

### Flow cytometry-based Ca^2+^ measurement

The FRET signal of parasites was measured by flow cytometry (Gallios^TM^, Beckman Coulter, Inc., Brea, CA). Before assay parasite-iRBCs were washed twice in phenol red- and plasma-free RPMI medium containing 0.5% AlbuMAX^®^ I. To measure CFP and FRET signals, iRBC were excited with a 405 nm laser and fluorescence was collected in the CFP channel with a standard 450/50 filter, while the FRET signal was measured with a 525/20 filter. To measure YFP signal, parasite-iRBCs were excited with a 488 nm laser and emission was taken with 525/20 filter. For each sample a minimum of five thousand YFP positive iRBCs were evaluated and non-infected RBCs were used as a baseline for signal detection. To measure the changes of cytosolic Ca^2+^, baseline Ca^2+^ levels were first measured for 60 sec for each sample, followed by addition of different Ca^2+^ inhibitors (CPA, TG and BHQ) for 3 min. FlowJo software (FlowJo LLC, OR, USA) was used to analyze the obtained data. The mean fluorescence intensities of the iRBC before and after adding inhibitors were obtained and the R_post_ ratio of iRBC after adding inhibitors were normalized by R_pre_ of iRBC before adding inhibitors to obtain the FRET signal changes of each inhibitor.

### Drug sensitivity assay

*P. falciparum* drug sensitivity was assessed using a SYBR^®^ Green I (Lonza Ltd, Basel, Switzerland), assay to determine IC_50_ using a protocol available at WorldWide Antimalarial Resistance Network (WWARN- http://www.wwarn.org/sites/default/files/INV08_PFalciparumDrugSensitivity.pdf).

### Homology modeling, docking simulation, and fragment molecular orbital calculation

The coordinates of the crystal structure of complex between rabbit SERCA and TG was downloaded from the Protein Data Bank (http://www.rcsb.org; 2AGV). A model structure of human SERCA and *Pf*SERCA were generated by a homology modeling based on the rabbit SERCA structure using Modeller9.14 (https://salilab.org/modeller/) 55 and PyMOL (http://www.pymol.org). Binding free energies of TG (PubChem CID: 446378) and dART (PubChem CID: 71939-50-9) with human/*Pf*SERCA were estimated by docking simulations using AutoDock4.2^56^. In these simulations, 200 individual genetic algorithm calculations were run in each of which 25 × 10^6^ energy evaluations were performed. Other model structures of TG and selected 144 amino acid residues located near the binding region were constructed by 2000 steps energy minimizations using AMBER99SB force field^57^. Using these structures, we performed fragment molecular orbital (FMO) calculations^58^ at second order Møller-Plesset perturbation theory with resolution of the identity approximation for analysis of van der Waals interactions. In these FMO calculations, cc-pVDZ basis set^59^ was employed and PAICS program^60^ was used.

### Statistical analyses

All statistical analysis was performed by Graphpad Prism 6 software (GraphPad Software, Inc. CA. USA).

## Additional Information

**How to cite this article**: Pandey, K. *et al.* Ca^2+^ monitoring in *Plasmodium falciparum* using the yellow cameleon-Nano biosensor. *Sci. Rep.* **6**, 23454; doi: 10.1038/srep23454 (2016).

## Supplementary Material

Supplementary Information
