# Supplementary material for: Ca2+ monitoring in Plasmodium falciparum using the yellow cameleon-Nano biosensor
Source: Sci Rep. 2016 Mar 23;6:23454. doi: 10.1038/srep23454 (PMC4804237; doi:10.1038/srep23454)
Supplement: Supplementary Information [file srep23454-s1.doc]

**Supplementary Figure**

**Ca^2+^ monitoring in *Plasmodium falciparum* using**

**the yellow cameleon-Nano biosensor**

Kishor Pandey, Pedro E. Ferreira, Takeshi Ishikawa, Takeharu Nagai, Osamu Kaneko & Kazuhide Yahata


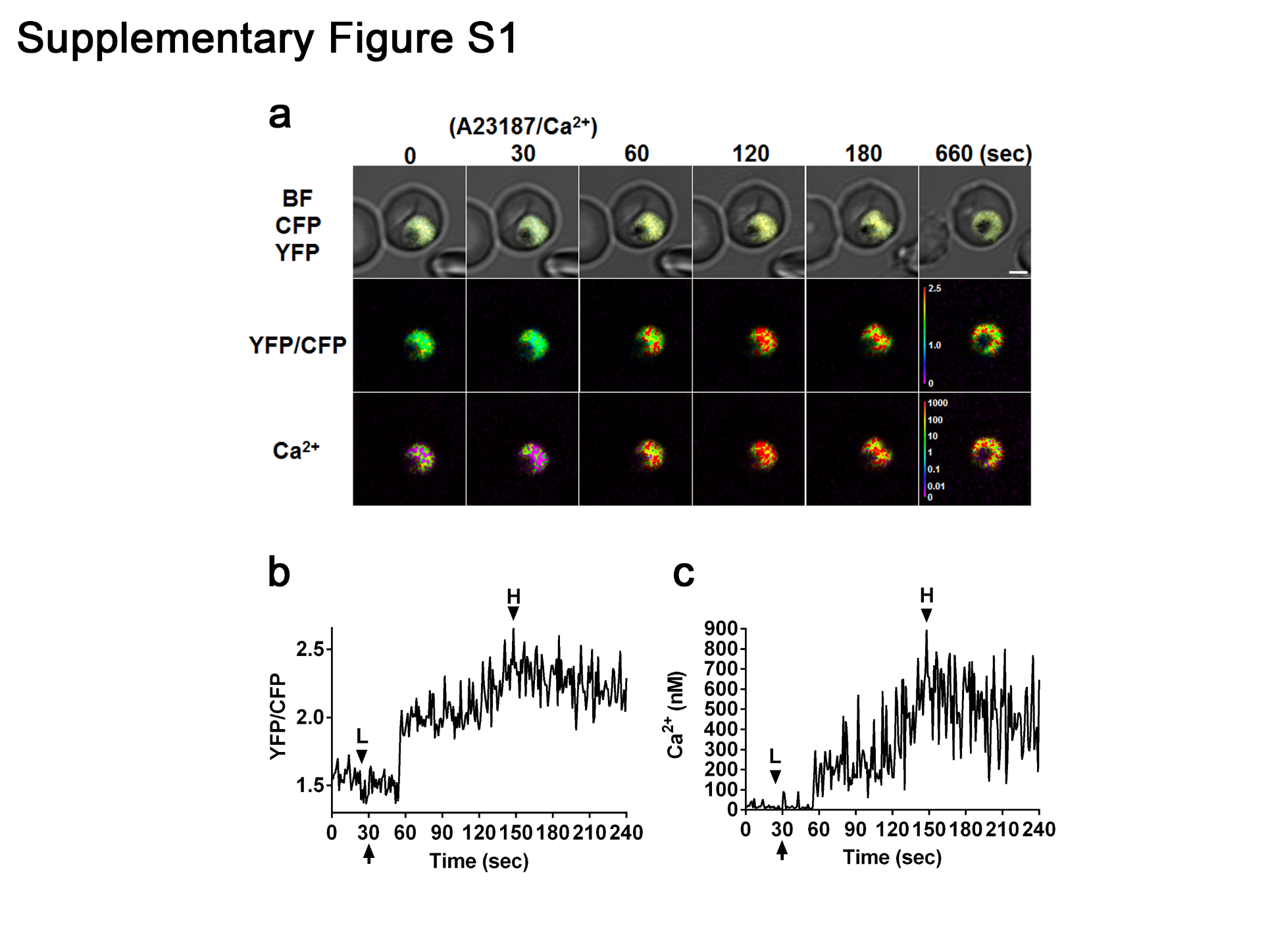


**Supplementary Figure S1. Determination of the cytosolic Ca^2+^ levels in *P. falciparum*.** (**a**) Images represent merged image (BF-CFP-YFP), FRET signal (YFP/CFP) and calculated Ca^2+^ concentration of the parasite cytosol at the trophozoite stage. The purple to red color scale represents low to high FRET efficiency (0 to 2.5) and 0 to 1000 nM Ca^2+^, respectively. Parasite cytosol where expressed YC-Nano50 is detected is selected as a region of interest for the FRET analysis. Scale bar, 2 µm. (**b** and **c**) Time course of the cytosolic Ca^2+^ level with the addition of 10 mM Ca^2+^ with A23187 at 30 sec shown with YFP/CYP value (**b**) or calculated Ca^2+^ concentration (**c**). The first arrowhead (28 sec) indicates the lowest (L) amount of Ca^2+^ before the addition of 10 mM Ca^2+^ with A23187 and the second arrowhead (148 sec) indicates the highest (H) amount of Ca^2+^ induced by the addition of 10 mM Ca^2+^ with A23187 (arrow). Cytosolic Ca^2+^ concentration is determined using the Grynkiewicz equation described in the Methods section.

**
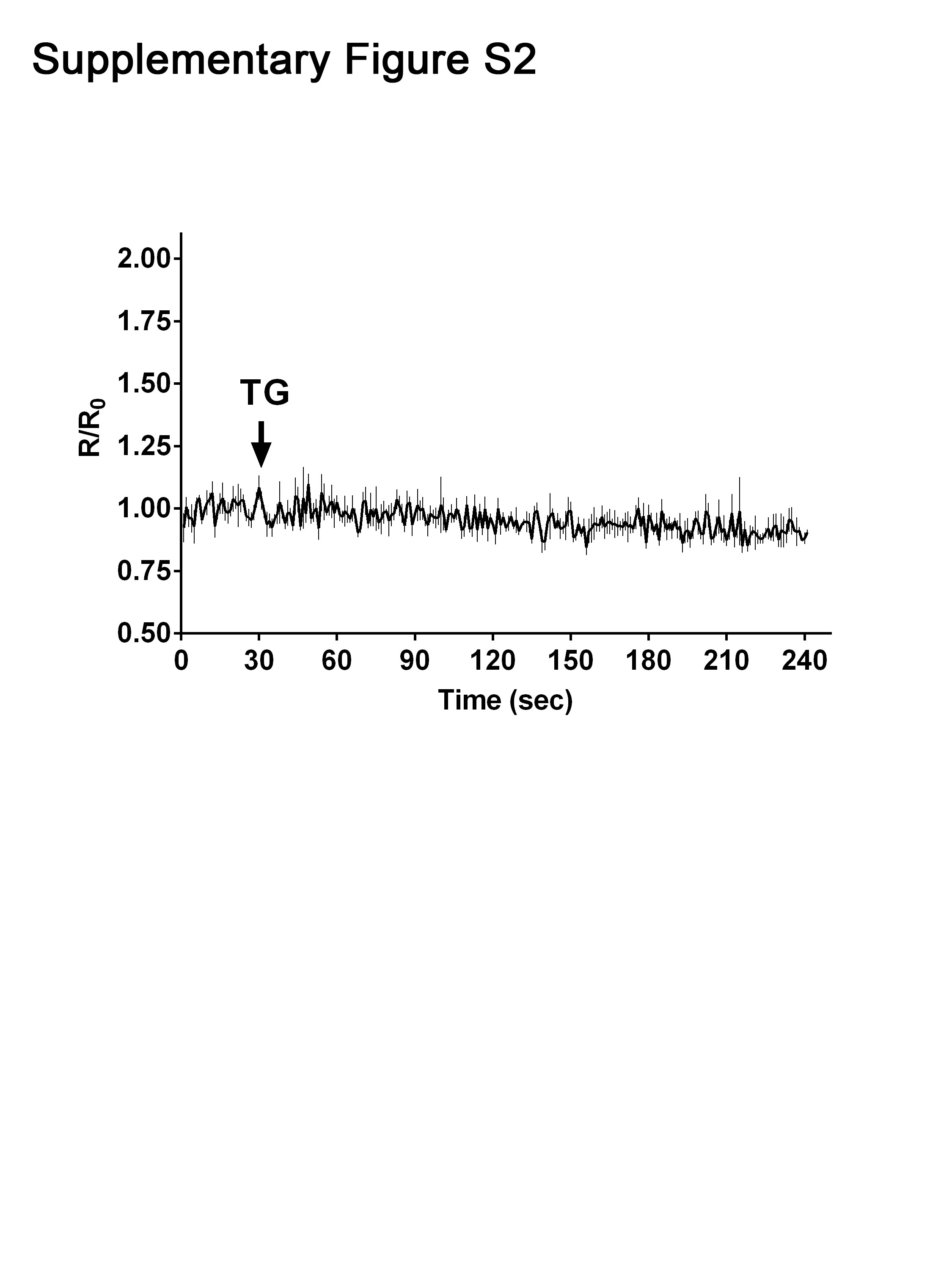
**

**Supplementary Figure S2. The effect of 76 µM** **thapsigargin (TG) on the cytosolic Ca^2+^ level in *P. falciparum*.** Time course of the cytosolic Ca^2+^ level with the addition of 76 µM TG at 30 sec (arrows). The traces are generated from the mean and standard error of the mean of 4 independent experiments.

**
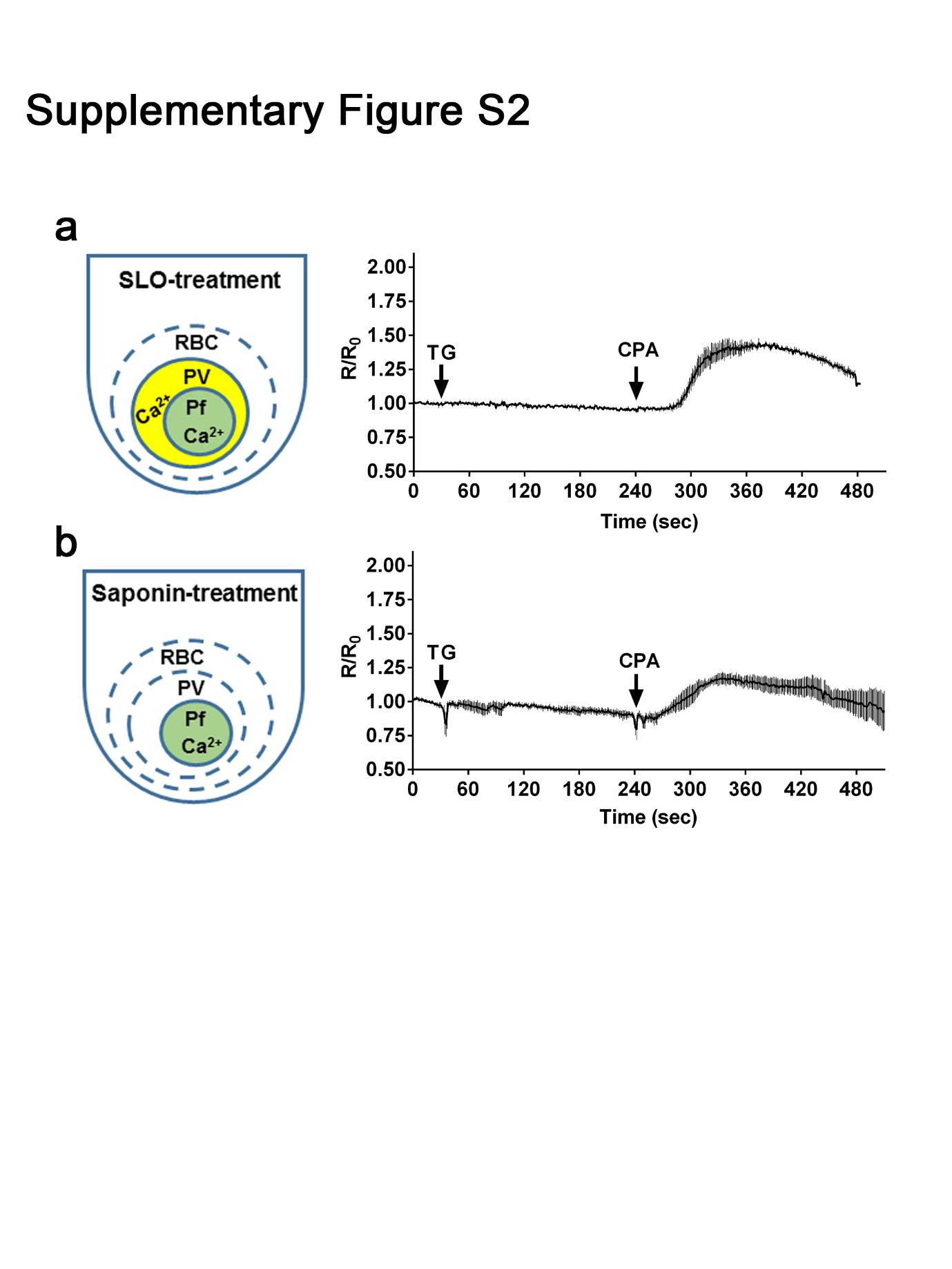
**

**Supplementary Figure S3. The effect of thapsigargin (TG) on the cytosolic Ca^2+^ level in *P. falciparum*.** Schematic indicates the status of the existence of Ca^2+^ in the different compartments in the parasite-infected red blood cell (iRBC) treated with streptolysin O (SLO) (**a**) or with saponin (**b**). Time courses of the cytosolic Ca^2+^ level with the addition of 1.5 µM thapsigargin (TG) at 30 sec and 3 µM cyclopiazonic acid (CPA) at 240 sec to SLO-treated iRBC in Ca^2+^ free medium (**a**) or to saponin-treated iRBC in Ca^2+^ free medium (**b**). The traces are generated from the mean and standard error of the mean of 3 independent experiments.

**
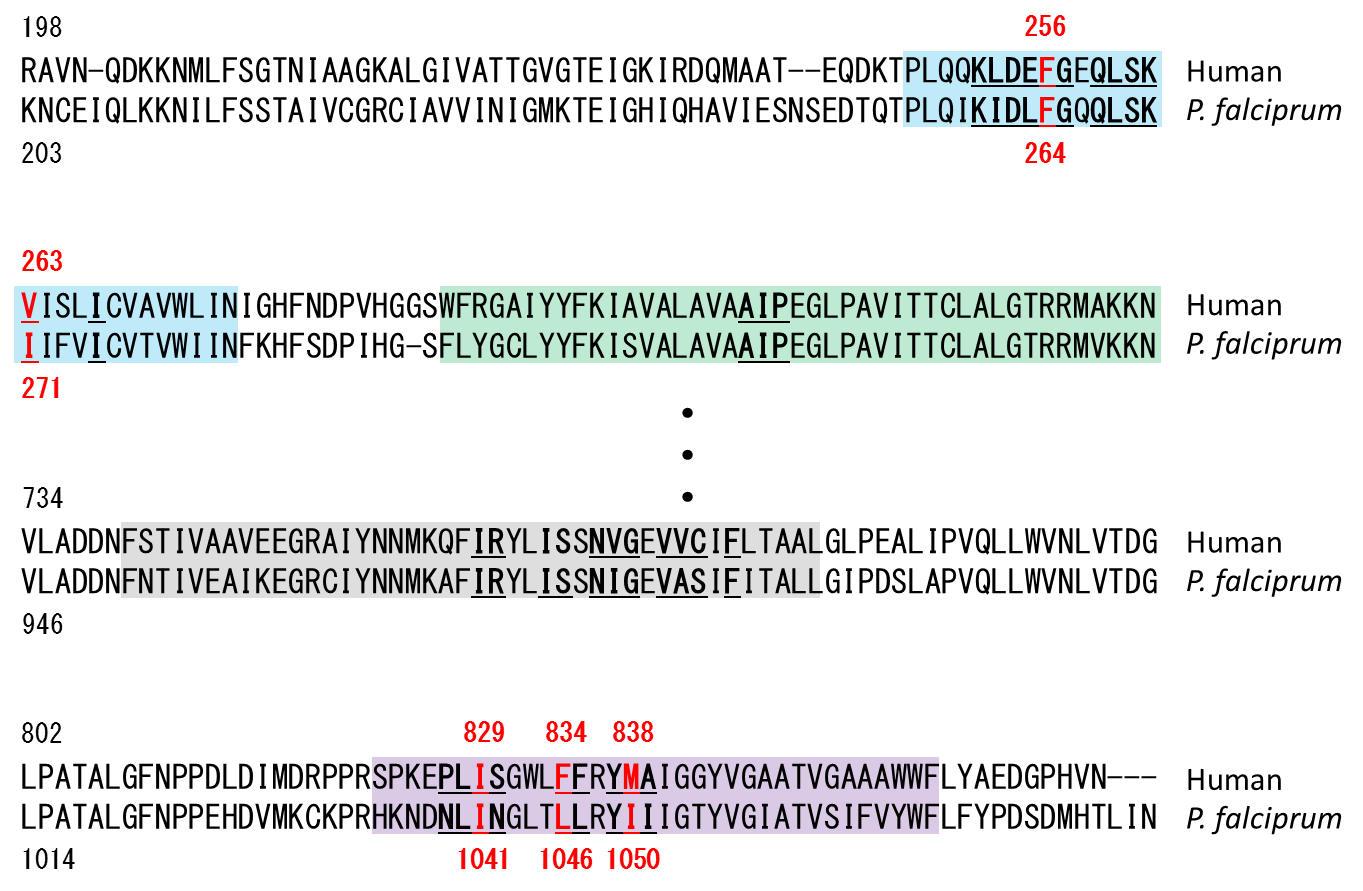
**

**Figure S4. Amino acid sequences of human and *P. falciparum* sarco/endoplasmic reticulum Ca^2+^-ATPase (SERCA) used for the calculation of the binding energy with thapsigargin (TG).** Amino acid sequences of human SERCA (*Hs*SERCA) and *P. falciparum* SERCA (*Pf*SERCA) are aligned and selected 144 amino acid residues structurally close to TG are shown with color (also in Figure 3 e–h). Thirty four amino acid residues located within 4.0 Å from TG in distance used to calculate the van der Waals interaction energies are bolded and underlined. Amino acid residues with red color appear to have impact on the difference in the binding energy and in the shape of the TG binding pocket between *Hs*SERCA and *Pf*SERCA (Fig. 3e–h and Supplementary Fig. S5).

**
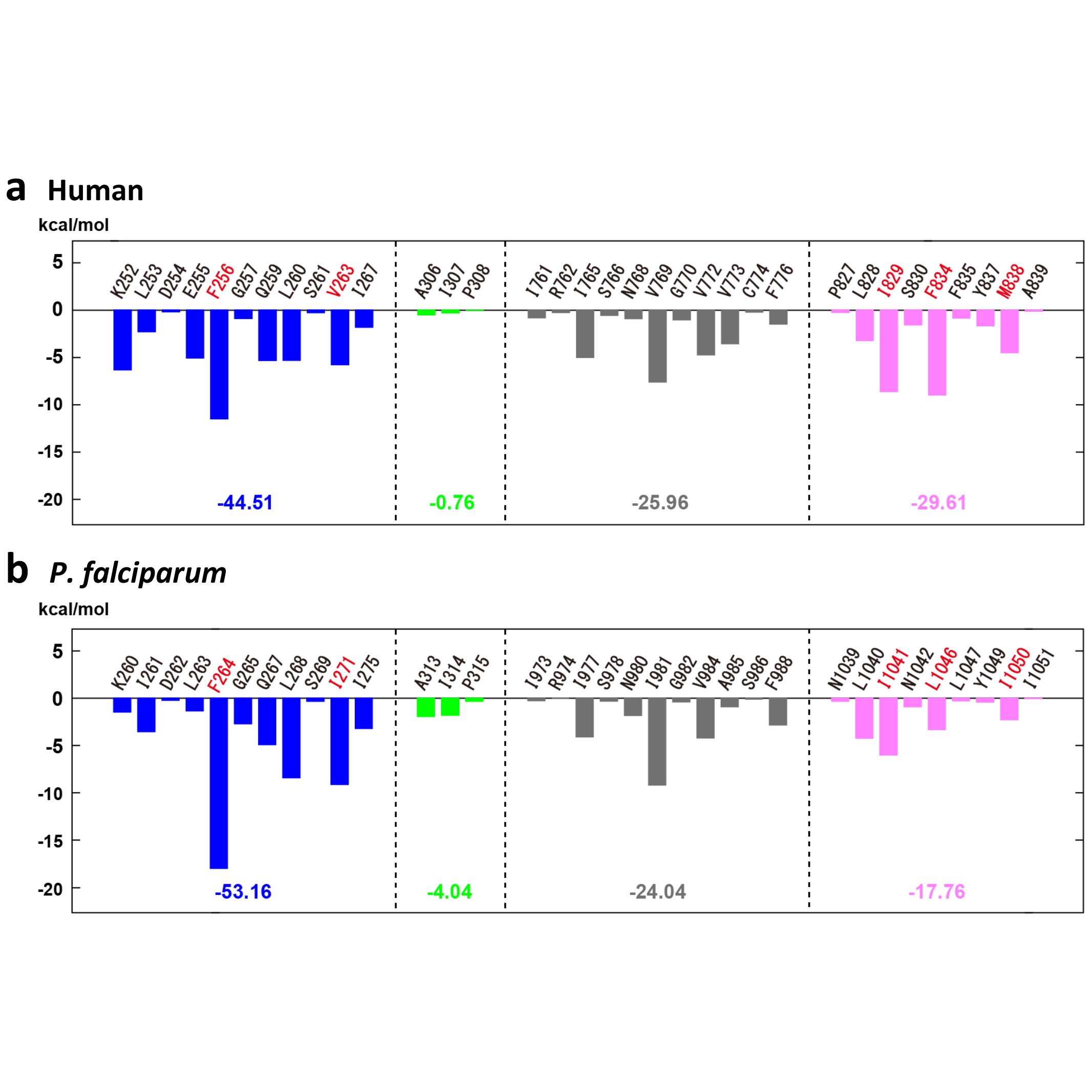
**

**Figure S5. Van der Waals interaction energies between thapsigargin (TG) and amino acid residues of human (a) and *P. falciparum* (b) SERCA.** Van der Waals interaction energies of 34 amino acid residues located within 4.0 Å from TG in distance are shown. These energies were calculated by the fragment molecular orbital method. A lower energy indicates more stable interaction. Color of the graph corresponds to Fig. 3e–h and Supplementary Fig S4.

**
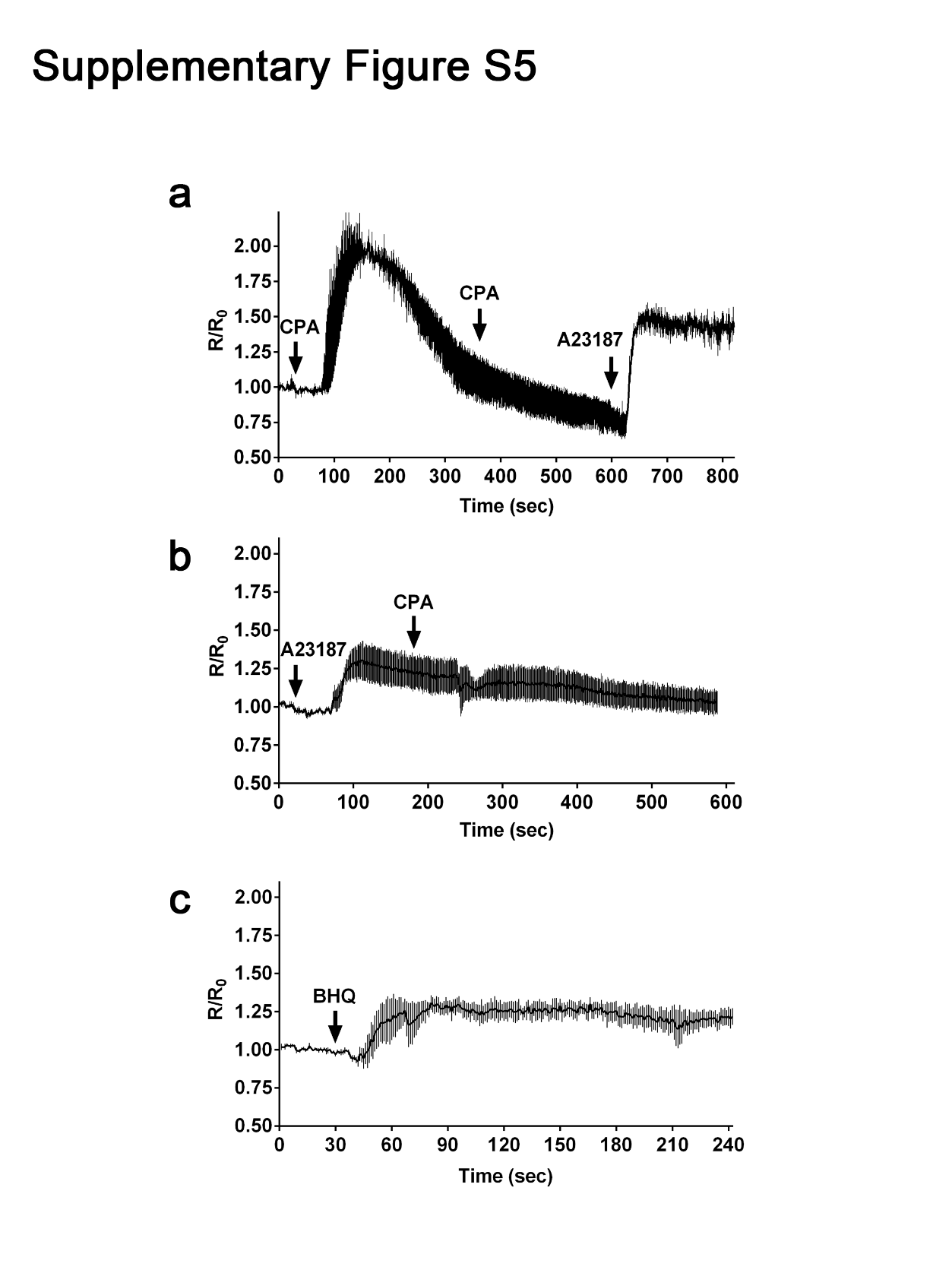
**

**Figure S6. Response of the cytosolic Ca^2+^ level in *P. falciparum* parasites against cyclopiazonic acid (CPA), A23187 calcium ionophore, and 2,5-Di-t-butyl-1,4-butylhydroquinone (BHQ).** (**a**) Time course of the cytosolic Ca^2+^ level with the addition of 15 µM CPA at 30 and 360 sec (arrows) and 10 µM A23187 at 600 sec (arrow). (**b**) Time course of the cytosolic Ca^2+^ level with the addition of 10 µM A23187 at 30 sec (arrow) and 15 µM CPA at 180 sec (arrow). (**c**) Time course of the cytosolic Ca^2+^ level with the addition of 2 µM BHQ at 30 sec (arrow). The traces are generated from the mean and standard error of the mean of 3 independent experiments.


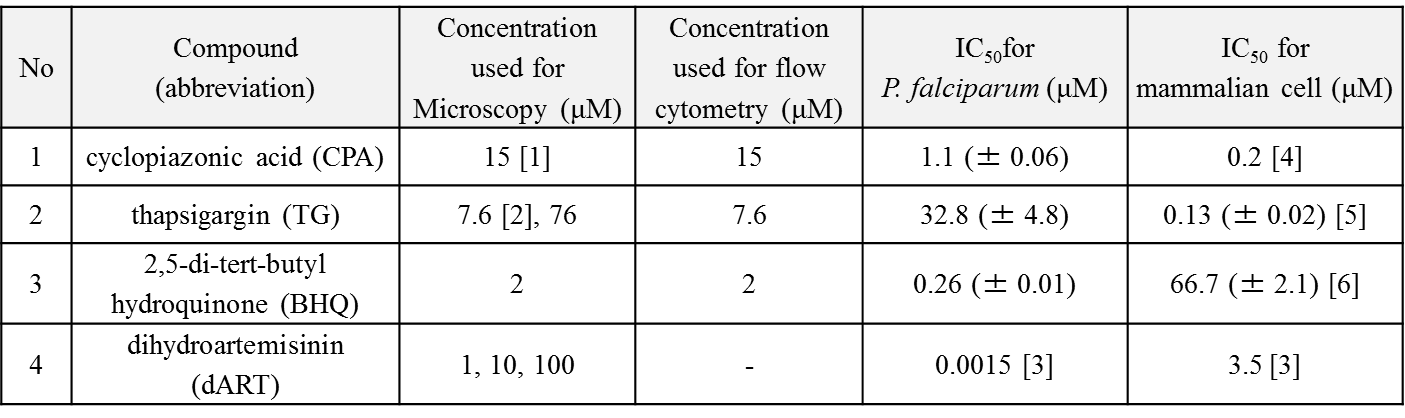


**Supplementary Table. Chemical compounds for monitoring on cytosolic Ca^2+^ level in *P. falciparum***

1. Laursen, M. et al. Cyclopiazonic acid is complexed to a divalent metal ion when bound to the sarcoplasmic reticulum Ca^2+^-ATPase. *J. Biol. Chem.* **284**: 13513-13518 (2009).
2. Varotti, F. P., Beraldo, F. H., Gazarini, M. L., Garcia, C. R. *Plasmodium falciparum* malaria parasites display a THG-sensitive Ca^2+^ pool. *Cell Calcium* **33**: 137-144 (2003).
3. Golenser, J., Waknine, J. H., Krugliak, M., Hunt, N. H., Grau, G. E. Current perspectives on the mechanism of action of artemisinins. *Int. J. Parasitol.* **36**:1427-1441 (2006).
4. Yard, N. J., Chiesi, M., Ball, H. A. Effect of cyclopiazonic acid, an inhibitor of sarcoplasmic reticulum Ca^2+^-ATPase, on the frequency-dependence of the contraction-relaxation cycle of the guinea-pig isolated atrium. *Br. J. Pharmacol.* **113**:1001-1007 (1994).
5. Fusi, F., Saponara, S., Gagov, H., Sgaragli, G. 2,5-Di-t-butyl-1,4-benzohydroquinone (BHQ) inhibits vascular L-type Ca^2+^ channel via superoxide anion generation. *Br. J. Pharmacol.* **133**:988-996 (2001).
6. Garavito-Aguilar, Z. V., Recio-Pinto, E., Corrales, A. V., Zhang, J., Blanck, T. J., Xu, F. Differential thapsigargin-sensitivities and interaction of Ca^2+^ stores in human SH-SY5Y neuroblastoma cells. *Brain Res.* **1011**:177-186 (2004).
